# Supplementary material for: The Small RNA Universe of Capitella teleta
Source: Front Mol Biosci. 2022 Feb 25;9:802814. doi: 10.3389/fmolb.2022.802814 (PMC8915122; doi:10.3389/fmolb.2022.802814)
Supplement: Supplementary file 1 [file DataSheet1.ZIP › Supplement/known/CAPTEscaffold_39_4063.pdf]

Provisional ID	:	CAPTEscaffold_39_4063
Score total	:	130603.6
Score for star read(s)	:	3.9
Score for read counts	:	130592.9
Score for mfe	:	2.2
Score for randfold	:	1.6
Score for cons. seed	:	3
Total read count	:	256164
Mature read count	:	255483
Loop read count	:	0
Star read count	:	681

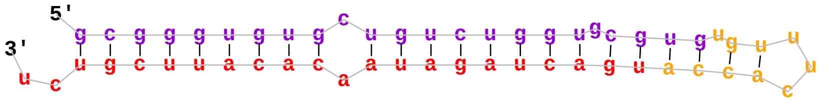

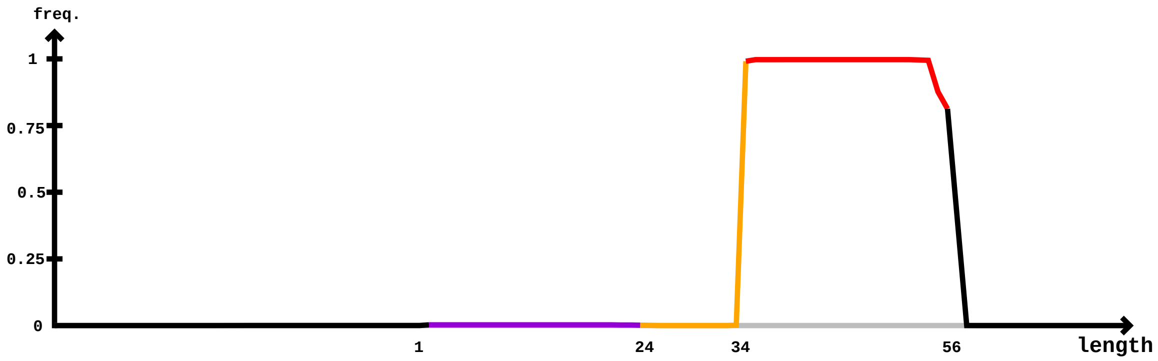

Star **Mature**

```
5'- ugugcguuuucacuguuggcugaugccauuggcggaggcgggugugcugucuggugcguguguuucaccaugacuagauaacacauucgucucugccucuugagcacccu  -3'   obs
    ugugcguuuucacuguuggcugaugccauuggcggaggcgggugugcugucuggugcguguguuucaccaugacuagauaacacauucgucucugccucuugagcacccu         exp
    ugugcguuuucacuguuggcugaugccauuggcggaggcgggugugcugucuggugcguguguuucaccaugacuagauaacacauucgucucugccucuugagcacccu         known
    .((((...........((((....))))..((((((((((((((((.((((((((.((((.((...)))))))))))))).))))))))))))))))......))))...   reads   mm   sample
    .........ucacuguuggcugaugccauuggcggag.........................................................................   1       0    seq
    ..........cacuguuggcugaugccauuggcggag.........................................................................   2       0    seq
    ...........acuguuggcugaugccauuggcggag.........................................................................   2       0    seq
    ..............guuggcugaugccauuggcggag.........................................................................   1       0    seq
    ..............guuggcugaugccauuggcUgag.........................................................................   1       1    seq
    ................uggcugaugccauuggcggag.........................................................................   21      0    seq
    .................ggcugaugccauuggcggag.........................................................................   6       0    seq
    ..................gcugaugccauuggcggag.........................................................................   7       0    seq
    .....................................gcgggugCgcugucuggugc.....................................................   1       1    seq
    .....................................gcgggugAgcugucuggugc.....................................................   1       1    seq
    .....................................gcgggAgugcugucuggugc.....................................................   1       1    seq
    .....................................gcgggugugcugucuggugc.....................................................   129     0    seq
    .....................................gcggguguAcugucuggugc.....................................................   1       1    seq
    .....................................gcgggugugcugucuggugcU....................................................   1       1    seq
    .....................................gcgggugugcugucuggugcg....................................................   7       0    seq
    .....................................gcgggugugcugucuggugcgu...................................................   181     0    seq
    .....................................gcgAgugugcugucuggugcgu...................................................   1       1    seq
    .....................................gcgggugugcugucuggugcgG...................................................   2       1    seq
    .....................................gcgggugugcuAucuggugcgu...................................................   1       1    seq
    .....................................gcgUgugugcugucuggugcgu...................................................   1       1    seq
    .....................................gcgggugugcugucuggugcguU..................................................   2       1    seq
    .....................................gcgggugugcCgucuggugcgug..................................................   1       1    seq
    .....................................gcgggugugcugucuggugcgGg..................................................   4       1    seq
    .....................................gcgggugugcuguUuggugcgug..................................................   1       1    seq
    .....................................gcAggugugcugucuggugcgug..................................................   3       1    seq
    .....................................gcgggugugcugucuggugcgug..................................................   299     0    seq
    .....................................gcgggugugcugucuggugcgugG.................................................   1       1    seq
    .....................................gcgggugugcugucuggugcgugu.................................................   14      0    seq
    .....................................gcgggugugcugucuggugcguguA................................................   1       1    seq
    .....................................gcgggugugcugucuggugcguguU................................................   25      1    seq
    .....................................gcgggugugcugucuggugcguguUu...............................................   1       1    seq
    ......................................cgggugugcugucuggugcgug..................................................   2       0    seq
```

Star **Mature**

ugugcguuuucacuguuggcugaugccauuggcggaggcgggugugcugucuggugcguguguuucaccaugacuagauaacacauucgucucugccucuugagcacccu			
....Cugacuagauaacacauucgu....................	100	1	seq
....augacuagauaacacauucgucu..................	6	0	seq
....Cugacuagauaacacauucgucu..................	199	1	seq
....Uugacuagauaacacauucgucu..................	1	1	seq
....ugacuagauaacacauuc......................	299	0	seq
....Ggacuagauaacacauuc......................	1	1	seq
....ugacuagauaaAacauuc......................	1	1	seq
....Agacuagauaacacauuc......................	2	1	seq
....ugacuagauaacacauuUg.....................	1	1	seq
....uAacuagauaacacauucg.....................	1	1	seq
....ugacuagauaacacauucC.....................	1	1	seq
....ugacuagauaacacauucg.....................	349	0	seq
....ugacuagauaacacauucU.....................	1	1	seq
....ugacuagauaaGacauucgu....................	1	1	seq
....ugacuagauaacGcauucgu....................	7	1	seq
....Agacuagauaacacauucgu....................	127	1	seq
....ugacuaAauaacacauucgu....................	6	1	seq
....ugacuaCauaacacauucgu....................	2	1	seq
....ugacuCgauaacacauucgu....................	1	1	seq
....ugacuagauaacaUauucgu....................	4	1	seq
....ugacuagauaacCcauucgu....................	1	1	seq
....ugacuagauaacacauAcgu....................	4	1	seq
....ugacuagaCaacacauucgu....................	3	1	seq
....ugacuagauNacacauucgu....................	1	1	seq
....ugacuagauaacacauCcgu....................	1	1	seq
....ugacuagGuaacacauucgu....................	3	1	seq
....ugaUuagauaacacauucgu....................	2	1	seq
....ugacuagauaacacUuucgu....................	1	1	seq
....ugacuagauUacacauucgu....................	2	1	seq
....ugacuagauaacaGauucgu....................	1	1	seq
....ugacuagauaacUcauucgu....................	9	1	seq
....ugacuagauGacacauucgu....................	6	1	seq
....ugacuagauaUcacauucgu....................	4	1	seq
....ugacuagUuaacacauucgu....................	2	1	seq
....uNacuagauaacacauucgu....................	1	1	seq
....ugacGagauaacacauucgu....................	4	1	seq
....ugacuagauCacacauucgu....................	1	1	seq
....ugacCagauaacacauucgu....................	2	1	seq
....ugUcuagauaacacauucgu....................	3	1	seq
....ugacuUgauaacacauucgu....................	4	1	seq
....ugacuagauaacaAauucgu....................	2	1	seq
....ugacuNgauaacacauucgu....................	2	1	seq
....ugacuagauaacacGuucgu....................	6	1	seq
....ugacuagauaacacaGucgu....................	3	1	seq
....ugacAagauaacacauucgu....................	40	1	seq
....ugacuagauaacacauucAu....................	5	1	seq
....uCacuagauaacacauucgu....................	6	1	seq
....ugacuagauaacacauuAgu....................	6	1	seq
....ugacuagauaacacauucgC....................	3	1	seq
....ugGcuagauaacacauucgu....................	4	1	seq
....ugacuagauaacacauuUgu....................	22	1	seq
....ugacuagauaaAacauucgu....................	5	1	seq
....ugacuagauaacacaCucgu....................	2	1	seq
....ugaAuagauaacacauucgu....................	6	1	seq
....ugacuagaGaacacauucgu....................	1	1	seq
....ugacuagauaacacaAucgu....................	19	1	seq
....ugacuaUauaacacauucgu....................	2	1	seq
....ugacuagauaacacauucgG....................	28	1	seq
....ugacuagaAaacacauucgu....................	11	1	seq
....ugaGuagauaacacauucgu....................	1	1	seq
....ugacuagauaacacauucgA....................	7	1	seq
....Ggacuagauaacacauucgu....................	18	1	seq
....ugacuagauaGcacauucgu....................	9	1	seq
....uUacuagauaacacauucgu....................	6	1	seq
....ugacuagauaacacauucUu....................	1	1	seq
....ugacuagauaacacauucgu....................	29227	0	seq
....Ngacuagauaacacauucgu....................	8	1	seq
....uAacuagauaacacauucgu....................	10	1	seq
....ugacuGgauaacacauucgu....................	4	1	seq
....Cgacuagauaacacauucgu....................	7	1	seq

```
                                    Star                            Mature
ugugcguuuucacuguuggcugaugccauuggcggaggcgggugugcugucuggugcguguguuucaccaugacuagauaacacauucgucucugccucuugagcacccu
......................................................................ugacuagauaaUacauucgu....................    9       1   seq
......................................................................ugUcuagauaacacauucguc...................    1       1   seq
......................................................................ugacuagauaacacauucguA...................    13      1   seq
......................................................................ugacuaAauaacacauucguc...................    3       1   seq
......................................................................ugacuagauaacaUauucguc...................    2       1   seq
......................................................................ugacCagauaacacauucguc...................    3       1   seq
......................................................................Ggacuagauaacacauucguc...................    15      1   seq
......................................................................ugacuagauaaAacauucguc...................    5       1   seq
......................................................................uAacuagauaacacauucguc...................    7       1   seq
......................................................................ugacuagauaGcacauucguc...................    7       1   seq
......................................................................ugacuagauaacaAauucguc...................    3       1   seq
......................................................................ugacuagauaacacauucgAc...................    3       1   seq
......................................................................Cgacuagauaacacauucguc...................    4       1   seq
......................................................................ugacuaUauaacacauucguc...................    2       1   seq
......................................................................ugacuagaAaacacauucguc...................    4       1   seq
......................................................................ugacuagauaacacUuucguc...................    2       1   seq
......................................................................ugacuagauaacacaAucguc...................    12      1   seq
......................................................................ugacuagauaacacauAcguc...................    7       1   seq
......................................................................ugacuagauaacacauucguc...................    16045   0   seq
......................................................................ugacuaCauaacacauucguc...................    1       1   seq
......................................................................ugacuagauaaGacauucguc...................    1       1   seq
......................................................................ugacuagauaUcacauucguc...................    6       1   seq
......................................................................ugacAagauaacacauucguc...................    19      1   seq
......................................................................ugacuagauaacacaGucguc...................    1       1   seq
......................................................................ugacuagauaacacauucguU...................    2       1   seq
......................................................................ugacuagUuaacacauucguc...................    1       1   seq
......................................................................ugacuagauaacacauucgCc...................    2       1   seq
......................................................................ugacuagauaacacauucUuc...................    2       1   seq
......................................................................ugaGuagauaacacauucguc...................    1       1   seq
......................................................................ugacuagauaacacauuGguc...................    2       1   seq
......................................................................ugacuagauGacacauucguc...................    2       1   seq
......................................................................ugacGagauaacacauucguc...................    4       1   seq
......................................................................ugacuagauaaUacauucguc...................    2       1   seq
......................................................................uCacuagauaacacauucguc...................    1       1   seq
......................................................................ugacuagauaacacauucAuc...................    6       1   seq
......................................................................ugacuCgauaacacauucguc...................    2       1   seq
......................................................................ugacuagauaacaGauucguc...................    2       1   seq
......................................................................Agacuagauaacacauucguc...................    66      1   seq
......................................................................ugacuagaCaacacauucguc...................    1       1   seq
......................................................................ugacuagGuaacacauucguc...................    3       1   seq
......................................................................ugacuagauaacacauuAguc...................    6       1   seq
......................................................................ugacuagauaacacauucgGc...................    1       1   seq
......................................................................ugacuagauaacacauucguG...................    1       1   seq
......................................................................ugacuGgauaacacauucguc...................    2       1   seq
......................................................................ugacuagauaacUcauucguc...................    1       1   seq
......................................................................Ngacuagauaacacauucguc...................    6       1   seq
......................................................................ugacuagauaacacauucCuc...................    1       1   seq
......................................................................ugacuagauaacacauuUguc...................    13      1   seq
......................................................................ugacuagauaacGcauucguc...................    2       1   seq
......................................................................ugacuagauaacacGuucguc...................    1       1   seq
......................................................................ugacuagauaacacaCucguc...................    2       1   seq
......................................................................ugaUuagauaacacauucguc...................    1       1   seq
......................................................................ugacuagauUacacauucguc...................    1       1   seq
......................................................................ugacuagauaacacauucgucA..................    204     1   seq
......................................................................ugacuagauaUcacauucgucu..................    22      1   seq
......................................................................ugacuagauaacCcauucgucu..................    6       1   seq
......................................................................ugacCagauaacacauucgucu..................    26      1   seq
......................................................................ugacuaUauaacacauucgucu..................    18      1   seq
......................................................................Ggacuagauaacacauucgucu..................    120     1   seq
......................................................................ugacGagauaacacauucgucu..................    13      1   seq
......................................................................ugacuagauaacacauGcgucu..................    8       1   seq
......................................................................ugacuagauaacaGauucgucu..................    13      1   seq
......................................................................ugacuagauaacacauAcgucu..................    65      1   seq
......................................................................ugacuagauaacacauucCucu..................    10      1   seq
......................................................................ugacuagauaCcacauucgucu..................    5       1   seq
......................................................................ugacuagauaacacaAucgucu..................    132     1   seq
......................................................................ugacuagauaacacauucguUu..................    27      1   seq
......................................................................ugacuagauUacacauucgucu..................    6       1   seq
......................................................................ugacuagauaacacauucguAu..................    27      1   seq
......................................................................ugacuagauaacacauuGgucu..................    13      1   seq
```

Star **Mature**

```
ugugcguuuucacuguuggcugaugccauuggcggaggcgggugugcugucuggugcgugguguuucaccaugacuagauaacacauucgucucugccucuugagcacccu
......................................................................ugacuagauaacacauucgAcu..................   47       1   seq
......................................................................ugacuagauaacaAauucgucu..................   17       1   seq
......................................................................ugacAagauaacacauucgucu..................   270      1   seq
......................................................................ugacuagauaacacauucgucG..................   622      1   seq
......................................................................ugaAuagauaacacauucgucu..................   30       1   seq
......................................................................ugacuagauaacacauucguGu..................   25       1   seq
......................................................................ugacuCgauaacacauucgucu..................   2        1   seq
......................................................................ugacuagCuaacacauucgucu..................   2        1   seq
......................................................................ugacuagGuaacacauucgucu..................   26       1   seq
......................................................................ugaUuagauaacacauucgucu..................   20       1   seq
......................................................................uNacuagauaacacauucgucu..................   12       1   seq
......................................................................ugCcuagauaacacauucgucu..................   4        1   seq
......................................................................ugacuagauaacaUauucgucu..................   17       1   seq
......................................................................ugacuagauaacacauuUgucu..................   157      1   seq
......................................................................uUacuagauaacacauucgucu..................   25       1   seq
......................................................................ugacuagauaacacUuucgucu..................   18       1   seq
......................................................................ugacuagauaacacauuAgucu..................   91       1   seq
......................................................................ugacuagauaacacaCucgucu..................   27       1   seq
......................................................................ugacuagauaacacauucAucu..................   31       1   seq
......................................................................ugacuUgauaacacauucgucu..................   20       1   seq
......................................................................ugacuagauaaUacauucgucu..................   14       1   seq
......................................................................Cgacuagauaacacauucgucu..................   27       1   seq
......................................................................uAacuagauaacacauucgucu..................   114      1   seq
......................................................................ugacuagauGacacauucgucu..................   40       1   seq
......................................................................ugacuagaGaacacauucgucu..................   6        1   seq
......................................................................ugUcuagauaacacauucgucu..................   20       1   seq
......................................................................Agacuagauaacacauucgucu..................   756      1   seq
......................................................................ugacuagaCaacacauucgucu..................   21       1   seq
......................................................................ugacuagUuaacacauucgucu..................   32       1   seq
......................................................................ugacuagauaacUcauucgucu..................   39       1   seq
......................................................................ugacuagauaacacauucgCcu..................   22       1   seq
......................................................................ugacuagauaacacauucgucu..................   202795   0   seq
......................................................................uCacuagauaacacauucgucu..................   24       1   seq
......................................................................ugacuagauaacacauCcgucu..................   10       1   seq
......................................................................ugGcuagauaacacauucgucu..................   28       1   seq
......................................................................ugaGuagauaacacauucgucu..................   13       1   seq
......................................................................ugacuNgauaacacauucgucu..................   4        1   seq
......................................................................ugacuagauaGcacauucgucu..................   81       1   seq
......................................................................ugacuGgauaacacauucgucu..................   45       1   seq
......................................................................Ngacuagauaacacauucgucu..................   61       1   seq
......................................................................ugacuagaAaacacauucgucu..................   51       1   seq
......................................................................ugacuagauaacacauucgucC..................   59       1   seq
......................................................................ugacuagauaacacauucUucu..................   6        1   seq
......................................................................ugacuagauaaAacauucgucu..................   82       1   seq
......................................................................ugacuagauaacacauucgGcu..................   21       1   seq
......................................................................ugacuagauaacacGuucgucu..................   36       1   seq
......................................................................ugacuagauaacGcauucgucu..................   39       1   seq
......................................................................ugacuaAauaacacauucgucu..................   85       1   seq
......................................................................ugacuaCauaacacauucgucu..................   9        1   seq
......................................................................ugacuagauaaGacauucgucu..................   7        1   seq
......................................................................ugacuagauaacacaGucgucu..................   20       1   seq
......................................................................ugacuagauaacacauucgucuA.................   193      1   seq
......................................................................ugacuagauaacacauucgucuc.................   7        0   seq
......................................................................ugacuagauaacacauucgucuU.................   72       1   seq
......................................................................ugacuagauaacacauucgucuG.................   9        1   seq
......................................................................ugacuagauaacacauucgucuUu................   18       1   seq
.......................................................................gacuagGuaacacauucgu....................   1        1   seq
.......................................................................gacAagauaacacauucgu....................   3        1   seq
.......................................................................gacuagauaacacauucgu....................   353      0   seq
.......................................................................gGcuagauaacacauucgu....................   4        1   seq
.......................................................................gacuagauaacacauucguc...................   154      0   seq
.......................................................................gacAagauaacacauucguc...................   1        1   seq
.......................................................................gacuagauaacacauuUguc...................   1        1   seq
.......................................................................gacuagauaacacauucguAu..................   2        1   seq
.......................................................................gacuagauaacacauucgucu..................   930      0   seq
.......................................................................gacuagauaacacauucgucG..................   3        1   seq
.......................................................................gacuagauaacacaAucgucu..................   3        1   seq
.......................................................................gacuagaAaacacauucgucu..................   1        1   seq
.......................................................................gGcuagauaacacauucgucu..................   7        1   seq
.......................................................................gacuagauaacUcauucgucu..................   1        1   seq
```

```
                                     Star                             Mature
ugugcguuuucacuguuggcugaugccauuggcggaggcgggugugcugucuggugcguguguuucaccaugacuagauaacacauucgucucugccucuugagcacccu
.......................................................................gacuaAauaacacauucgucu..................   1    1    seq
.......................................................................gacuagauaacaUauucgucu..................   1    1    seq
.......................................................................gacuagauaacacauuAgucu..................   4    1    seq
.......................................................................Uacuagauaacacauucgucu..................   2    1    seq
.......................................................................gacAagauaacacauucgucu..................   6    1    seq
.......................................................................gacuagauaacacauucgucA..................   2    1    seq
.......................................................................gacuagauaacacauuUgucu..................   1    1    seq
.......................................................................gacuagauaacacauucgucuA.................   1    1    seq
........................................................................acuagauaacacauucgucu..................   5    0    seq
.........................................................................cuagauaacacauucgucu..................   2    0    seq
..........................................................................uagauaacacauucgucu..................   2    0    seq
.............................................................................auaacacauucgucucugccu............   1    0    seq
............................................................................................cugccucuugagcacccu   5    0    seq
............................................................................................cugccucuugagcacccA   2    1    seq
```
